# Resveratrol: French Paradox Revisited

**DOI:** 10.3389/fphar.2012.00141

**Published:** 2012-07-17

**Authors:** Betul Catalgol, Saime Batirel, Yavuz Taga, Nesrin Kartal Ozer

**Affiliations:** ^1^Department of Biochemistry, Faculty of Medicine, Genetic and Metabolic Diseases Research Center, Marmara UniversityIstanbul, Turkey

**Keywords:** cancer, cardiovascular diseases, resveratrol, signal transduction

## Abstract

Resveratrol is a polyphenol that plays a potentially important role in many disorders and has been studied in different diseases. The research on this chemical started through the “French paradox,” which describes improved cardiovascular outcomes despite a high-fat diet in French people. Since then, resveratrol has been broadly studied and shown to have antioxidant, anti-inflammatory, anti-proliferative, and anti-angiogenic effects, with those on oxidative stress possibly being most important and underlying some of the others, but many signaling pathways are among the molecular targets of resveratrol. In concert they may be beneficial in many disorders, particularly in diseases where oxidative stress plays an important role. The main focus of this review will be the pathways affected by resveratrol. Based on these mechanistic considerations, the involvement of resveratrol especially in cardiovascular diseases, cancer, neurodegenerative diseases, and possibly in longevity will be is addressed.

## Introduction

The “French paradox” is a term that was generated in 1992 based on epidemiological data from French people who had a low incidence of coronary heart diseases (CHD) despite the consumption of a diet in high saturated fat. This prompted researchers to think about a possible reason, and led Renaud and de Lorgeril (1992) to propose that moderate wine consumption (almost 57% of the overall alcoholic drink consumption in France) explained this apparent discrepancy, and further suggesting that a decrease in platelet aggregation may be the main factor of the effect on CHD. In an investigation by the World Health Organization, the ratio of CHD-related mortality was found to be two- to threefold lower in France compared to other countries such as the USA, UK, and Sweden (World Health Organization, 2009). Additionally, a study was carried out in Copenhagen with 6051 men and 7234 women aged 30–70 years. In this study, low to moderate wine intake was associated with lower mortality from cardiovascular and cerebrovascular diseases (Gronbaek et al., 2001). After these observations, a great deal of attention has been paid to the French paradox and thousands of studies have been performed on different aspects.

The French paradox has evolved considerably since 1992. Several studies indicate that heavy wine drinkers may have almost the same mortality rate due to CHD. Heavy alcohol consumption also increases the prevalence of myocardial infarction, cardiomyopathy, cardiac arrhythmias, hypertension, hemorrhagic shock, and sudden death (Djousse et al., 2002; Lucas et al., 2005), confirming that heavy alcohol consumption is harmful to the cardiovascular system. However, the correlation of the drinking patterns with a higher cardiovascular risk apparently depends on the type of drink (van de Wiel and de Lange, 2008).

Many compounds have been identified to be specifically abundant in red wine and many of these have a phenolic structure. The main compounds in red wine (Waterhouse, 2002) include flavonols such as myricetin, kaempferol, and the predominant quercetin, the flavan-3-ol monomers catechin and epicatechin, the oligomeric, and polymeric flavan-3-ols, or proanthocyanidins, anthocyanins, phenolic acids including gallic acid, caftaric acid, caffeic acid, *p*-coumaric acid, and the stilbene resveratrol. Thus, red wine appears to be the richest source of resveratrol through the skins, seeds, and stems of the grapes that are used.

## Resveratrol

Resveratrol is a stilbenoid named *trans*-3,4′,5,-trihydroxystilbene and consists of two aromatic rings which are attached by a methylene bridge. It is a natural phenol and phytoalexin, produced naturally by 72 different plant species especially grapevines, pines, and legumes (Soleas et al., 1997). It is also present in peanuts, soy beans, and pomegranates in high concentrations. In particular Botrytis cinerea infection in grapes leads to the exclusive synthesis of resveratrol in the leaf epidermis and grape skins. Since grape skins are not fermented during white wine production, only red wines contain noticeable amounts of resveratrol. This compound was first mentioned by M. Takaoka in 1939 after its isolation from the root of the white hellebore, *Veratrum grandiflorum*. The name resveratrol was derived from this source since it is a resorcinol derivative from a *Veratrum* species (Lancon et al., 2007).

Resveratrol is present in *cis*/*trans* isoforms both of which may be glucosylated and the major *trans* isomer is the biologically active one. Resveratrol is also produced by chemical (Farina et al., 2006) and biotechnological (Trantas et al., 2009) synthesis and sold as a nutritional supplement following its derivation from Japanese knotweed which is the Itadori plant (*Polygonum cuspidatum*; Kimura et al., 1985).

Resveratrol was identified to be converted to its monohydroxylated form, piceatannol, by the cytochrome P450 enzyme CYP1B1 (Potter et al., 2002). Piceatannol possesses stronger antitumor effects than resveratrol itself, and its hydroxyl group was shown to increase its inhibitory activity on protein kinases (Thakkar et al., 1993) and nuclear factor kappa B (NFκB; Ashikawa et al., 2002) and its inducer activity on apoptosis (Wieder et al., 2001). Also the actions of resveratrol were found to be related to its 3-*O*-sulfate (predominantly) and 3-*O*-beta-d-, 4′-*O*-beta-d-glucuronide metabolites (Kaldas et al., 2003). Resveratrol was found to be metabolized mainly to its glucuronide metabolite in the rat small intestine used as a model (Lancon et al., 2004). However, in human plasma resveratrol sulfate was found to be the major metabolite upon oral ingestion of resveratrol (Walle et al., 2004). This sulfation is thought to be the main limiting factor for the bioavailability of the compound. Sulfates were also found to be the predominant resveratrol metabolites in Caco-2 human colon carcinoma cells but this formation could be inhibited by resveratrol itself (Maier-Salamon et al., 2006). Sulfotransferase 1A1 is found to be responsible for the formation of 3-*O*-sulfates, and these metabolites show reduced anticancer activity in human breast cancer cells compared to resveratrol (Murias et al., 2008). In order to reduce the fast metabolism of resveratrol the stability of resveratrol can be improved by liposome-encapsulation (Coimbra et al., 2011).

Resveratrol bioavailability is believed to be affected by the food matrix. Human subjects were administered pure resveratrol, and high amount of glucuronide metabolites were detected in the plasma and urine. However, oral doses of grape juice also resulted in glucuronide and sulfate conjugates in the human plasma and urine (Meng et al., 2004). The presence of resveratrol glucosides in grape juice following glycosylation suggests a lower bioavailability compared to the pure resveratrol is due to the role of matrix sugar. But the administration of polyphenols in different vehicles (white wine, grape juice, and vegetable juice) did not show any difference in terms of the absorption of total resveratrol (Goldberg et al., 2003). However, food matrix-related factors such as proteins, carbohydrates, fiber, fat, alcohol have been suggested as positive or negative modifiers of the absorption of polyphenols (Azuma et al., 2002; Visioli et al., 2003). In a pharmacokinetic study of resveratrol carried out with 11 healthy volunteers, resveratrol was found to be better absorbed from natural grape products than from tablets, confirming the importance of the matrix to its bioavailability (Ortuno et al., 2010).

**Figure 1:**
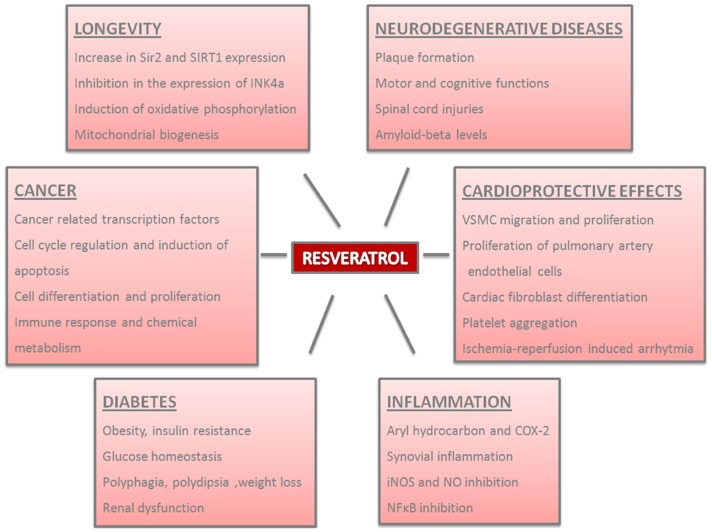
**Physiological and pathological effects of resveratrol in the organisms**. Resveratrol plays important roles in distinct processes and the parameters of these effects are summarized.

Resveratrol is a multipurpose compound believed to be effective in improving health and preventing or treating chronic diseases, therefore it has been the focus of many animal and human studies (Figure 1). Its biological activities have been shown to depend on its structural determinants including the number and position of carboxyl groups, intramolecular hydrogen bonding, stereoisomery, and the presence of double bond. *Trans*-stilbene compounds which possess ortho-diphenoxyl or para-diphenoxyl functionalities having a 4′-hydroxyl group and double bond show high chemopreventive activity (Ovesna and Horvathova-Kozics, 2005). Coppa et al. (2011) synthesized resveratrol analog 4,4′-dihydroxy-*trans*-stilbene with two hydroxyl groups in the 4 and 4′ position to obtain a molecule more active than resveratrol; this analog was more active than resveratrol in the inhibition of secretion of the potent vasoconstrictor peptide, endothelin-1.

To increase the activity, several resveratrol analogs have been synthesized. Researchers have investigated the structure-activity relationship by changing the number and the position of hydroxyl groups (Szekeres et al., 2010). Increasing the number of hydroxyl groups on the phenol rings was proved to inhibit the growth of human tumor cells. Also, 3,3′,4′,5-tetrahydroxy-*trans*-stilbene, 3,4,4′,5-tetrahydroxy-*trans*-stilbene and 3,3′,4,4′,5,5′-hexahydroxy-*trans*-stilbene showed almost 7000-fold higher antiradical activity compared to resveratrol (Murias et al., 2004, 2005). A hydroxyl group at the meta position in another analog 3,5,3′,4′,5′-pentahydroxy-*trans*-stilbene was identified as crucial for the inhibition of cell transformation (Lee et al., 2008). Hexahydroxystilbene, M8, was identified as the most effective analog in various tumor cell lines with its high free radical scavenging and therefore antitumor and antimetastatic activities. Whether the same structure-activity relationships apply to each of the effects of resveratrol, remains to be elucidated.

## Affected Molecular and Cellular Mechanisms on the Cardiovascular Cells

Many *in vitro* studies have been carried out to elucidate the mechanisms of the action of resveratrol. Oxidative damage and reactive species (RS) are strongly implicated in the pathogenesis of cardiovascular diseases (Park et al., 1991; Repine, 1991; Ago et al., 2010; Schiffrin, 2010). Free radicals include RS such as reactive oxygen species (ROS) and reactive nitrogen species (RNS). RS can damage cellular components such as proteins, lipids, carbohydrates, and nucleic acids (Nordberg and Arner, 2001). Because of the role of oxidative stress in cardiovascular diseases, a great deal of attention has been focused on natural antioxidants in the treatments.

The main ROS implicated in cardiovascular diseases are superoxide (${\text{O}}_{\text{2}}^{•\text{–}}$), hydroxyl (${\text{OH}}^{•}$), and hydrogen peroxide (H_2_O_2_) and the RNS are nitric oxide (NO) and peroxynitrite. While superoxide and hydroxyl radicals are more reactive, hydrogen peroxide is more membrane permeable. Concerning the basic mechanism, these oxygen species are converted to each other by several mechanisms. ${\text{O}}_{\text{2}}^{•\text{–}}$ is dismutated non-enzymatically or enzymatically by superoxide dismutase (SOD) to H_2_O_2_. Also various enzymes located in the plasma membrane, the cytosol, peroxisomes, and mitochondria catalyze ROS formation. Resveratrol seems to increase vascular oxidative stress resistance by scavenging H_2_O_2_ and preventing oxidative stress-induced endothelial cell death and it has been proposed that the antioxidant and anti-apoptotic effects of resveratrol are responsible, at least in part, for its cardioprotective effects (Ungvari et al., 2007). Resveratrol can also inhibit the formyl methionyl leucyl phenylalamine (fMLP) induced production of ROS from monocytes correlated with significant inhibitory effects on fMLP-induced phosphatidylinositol 3-kinase (PI3K) activity and Akt phosphorylation (Poolman et al., 2005). It was shown that resveratrol attenuates increase in ROS induced by oxidized low density lipoproteins (oxLDL) and H_2_O_2_ levels in bovine aortic smooth muscle cells (Liu and Liu, 2004).

Nitric oxide is one of the important RNS in the pathogenesis of cardiovascular diseases. NO is classified as a free radical in terms of its unpaired electron but since it is not able to initiate typical damage reactions to biomolecules it is relatively a non-reactive radical. NO is produced *in vivo* during the oxidation of one of the terminal guanidino-nitrogen atoms of l-arginine (Palmer et al., 1988) to l-citrulline catalyzed by NO synthase (NOS), in the presence of nicotinamide adenine dinucleotide phosphate (NADPH) and O_2_ (Moncada et al., 1991; Griffith and Stuehr, 1995). It is produced by the endothelial NOS (eNOS) and is a key determinant of cardiovascular homeostasis (in endothelial cells not in all cell types). Of note, low concentrations of NO are considered to be beneficial in the cardiovascular system, e.g., by causing vasodilatation, and only high concentrations are thought to have negative effects due to reactive properties. Incubation of human umbilical vein endothelial cells (HUVEC) and HUVEC-derived EA.hy 926 cells with resveratrol upregulated the expression of eNOS mRNA. The expression of eNOS protein and the production of eNOS-derived NO were also increased after long-term incubation with resveratrol. This stimulation of eNOS expression and activity may contribute to the cardiovascular protective effects attributed to resveratrol (Wallerath et al., 2002). A significant decrease in intracellular NO level and superoxide overproduction was found in HUVEC treated with oxLDL, but not with LDL; this redox imbalance was prevented by the addition of quercetin or resveratrol (Kostyuk et al., 2011).

Resveratrol, with the aromatic groups in its structure, is able to function as antioxidant and prevent oxidation reactions. Resveratrol has been shown to have capacity to sequester 2,2-azinobis(3-ethylbenzthiazoline-6-sulfonic acid; ABTS), 1,1-diphenyl-2-picrylhydrazyl (DPPH), and to scavenge hydroxyl radical (Soares et al., 2003). With its antioxidant capacity, resveratrol was shown to delay oxidative stress related apoptosis in several cell types including peripheral blood mononuclear cells, human retinal pigment epithelium cells, rat pheochromacytoma cells, and mouse 3T3 fibroblasts (Jang and Surh, 2001; Losa, 2003; Kutuk et al., 2004). The antioxidative property of resveratrol could make this compound protective in atherosclerosis since LDL oxidation is an important process in this disease. *In vitro* results showed that resveratrol inhibits copper and 2,2′-azobis (2-amidinopropane) dihydrochloride (AAPH) induced LDL oxidation (Belguendouz et al., 1997; Fremont et al., 1999). Blood platelets may also generate ROS at the site of atherosclerotic lesion, and resveratrol was shown to inhibit this ROS production and decrease the degree of lipid peroxidation (Olas and Wachowicz, 2002). The results of Miura et al. (2000) suggested that resveratrol exerts its scavenging activity of lipid peroxidation via lipid peroxyl and/or carbon centered radicals. On the other hand, resveratrol at low concentrations, was shown to elicit a pro-oxidant property by an increase in intracellular superoxide production (Ahmad et al., 2003). It was also a pro-oxidant in the presence of copper ions (Ahmad et al., 2005).

**Figure 2:**
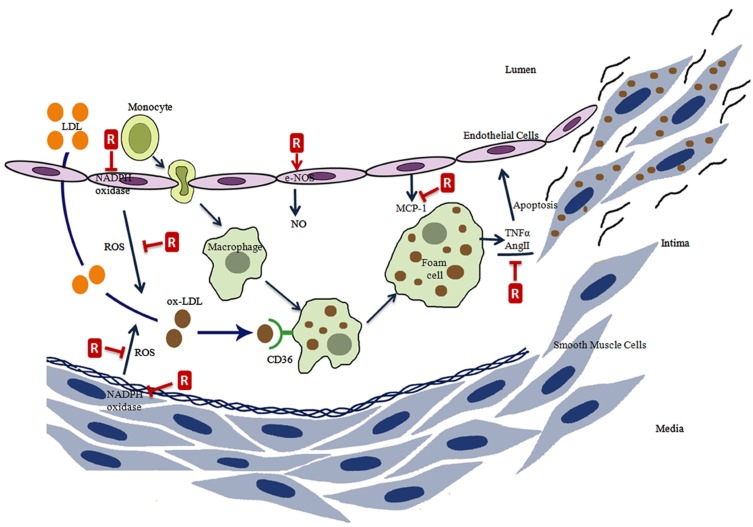
**The effects of resveratrol on atherosclerosis process**. Resveratrol (R) prevents the ROS formation directly or by the inhibition of NADPH oxidase, induces eNOS activity, inhibits monocyte chemoattractant protein-1 (MCP-1) expression, inhibits the tumor necrosis factor alpha (TNFα), and angiotensin II (AngII) induced endothelial cell apoptosis.

Resveratrol has been shown to be cardioprotective in many conditions (Figure 2; Gresele et al., 2011). Cardiac fibroblasts regulate myocardial remodeling by proliferating, differentiating, and secreting extracellular matrix proteins. Prolonged activation of cardiac fibroblasts leads to cardiac fibrosis and reduced myocardial contractile function. Resveratrol was tested on the growth and proliferation of cardiac fibroblasts and their differentiation to the hypersecretory myofibroblast phenotype. Cardiac fibroblasts pretreated with resveratrol showed inhibition of basal and angiotensin (ANG) II-induced extracellular signal-regulated kinase (ERK) 1/2 and ERK kinase activation. This inhibition reduced basal proliferation and blocked ANG II-induced growth and proliferation of cardiac fibroblasts (Olson et al., 2005). Cardiac troponin, a heterotrimeric protein complex that regulates heart contraction, represents an attractive target for the development of drugs for treating heart disease. Resveratrol was identified as modulating troponin C–troponin I interactions (Pineda-Sanabria et al., 2011).

Atherosclerosis is a wide ranging cardiovascular disease and is characterized by the proliferation of certain cell types within the arterial wall. This proliferation results from the accumulation of cholesterol and triglyceride carrying plasma lipoproteins in the arteries (Stocker and Keaney, 2004). In the disease process, phagocytic monocytes are transformed into macrophage foam cells following penetration into the subendothelial space. Oxidatively modified lipoproteins are endocytosed by a receptor-mediated mechanism and these foam cells appear to be loaded with lipid droplets rich in cholesteryl esters (Osterud and Bjorklid, 2003; Schmitz and Grandl, 2007). CD36 takes the most important place among the receptors responsible for the uptake of lipoproteins (Stocker and Keaney, 2004; Schmitz and Grandl, 2007). Foam cells are formed due to an accumulation of lipoproteins in macrophages and smooth muscle cells. Thus visible lesions in the pathogenesis of atherosclerosis originate mainly from foam cells and from an adaptive thickening of the intima (Steinberg, 2009).

Resveratrol significantly affected the proliferation and the shape of bovine pulmonary artery endothelial cells; growth was suppressed and cells became elongated. The cellular phenotype induced by resveratrol was dependent on intracellular calcium and tyrosine kinase activities, and on the assembly of actin microfilaments and microtubules, but was unrelated to protein kinase C activity. Resveratrol treatment also resulted in an increase in phosphorylated ERK1/2 and NOS expression (Bruder et al., 2001). Resveratrol inhibited the proliferation of pulmonary artery endothelial cells, which, based on a flow cytometric analysis, correlates with the suppression of cell progression through the S and G2 phases of the cell cycle (Hsieh et al., 1999).

Lipid peroxidation and LDL oxidation are the early events in atherosclerotic lesion formation (Salvayre et al., 2002; Vogiatzi et al., 2009). The source of ROS formation in the vessel wall are thought to be mostly macrophages but also other cells like endothelial, smooth muscle, and adventitial cells produce RS in the vessel wall (Fortuno et al., 2005). In addition, polymorphonuclear leukocytes (PMN) are thought to contribute to the pathogenesis of acute CHD together with the RS production. Resveratrol exerted a strong inhibitory effect on ROS produced by PMN stimulated with fMLP and the results obtained from this study indicated that *trans*-resveratrol interferes with the release of inflammatory mediators by activating PMN and down-regulating adhesion-dependent thrombogenic PMN functions (Rotondo et al., 1998).

Hypercholesterolemia is a major risk for coronary artery diseases and ROS have also been implicated in the development of hypercholesterolemic atherosclerosis (Stokes et al., 2002). Patients with elevated cholesterol may have increased susceptibility to Alzheimer’s disease in addition to coronary artery disease and hypertension (Pappolla et al., 2003) and amyloid beta formation is known to be induced by cholesterol. In relation to its effect on cholesterol homeostasis, resveratrol also enhances the cholesterol efflux mediated by apolipoprotein A1 (apoA-1) up-regulating the ATP binding cassette transporter A1 (ABCA-1) receptors, and reducing cholesterol influx or uptake in J774 macrophages (Berrougui et al., 2009).

Key components in atherogenesis are known to be increased by RS in the vascular endothelium. They include signaling molecules such as redox sensitive transcription factor NFκB activation and adhesion molecules such as selectins, vascular cell adhesion molecule-1 (VCAM-1), intercellular adhesion molecule-1 (ICAM-1), and chemokines such as monocyte chemoattractant protein-1 (MCP-1). Expression of adhesion molecules and MCP-1 are also key steps for the monocyte adhesion and migration to form macrophages and foam cells. Macrophage colony-stimulating factor (M-CSF) is an important factor regulating the survival, proliferation, differentiation, and chemotaxis of macrophages (Fan and Watanabe, 2003; Pixley and Stanley, 2004; Chitu and Stanley, 2006; Harizi and Gualde, 2006). Several studies have been carried out with endothelial cells. Resveratrol is known to be a tyrosine kinase inhibitor like other members of the tyrphostin family and at concentrations as low as 1 μM and 100 nM, it significantly inhibits ICAM-1 and VCAM-1 expression by tumor necrosis factor α (TNF)-stimulated HUVECs and lipopolysaccharide (LPS)-stimulated human saphenous vein endothelial cells (HSVEC), respectively. Resveratrol also significantly inhibited the adhesion of neutrophils to TNF-α-stimulated NIH/3T3 ICAM-1-transfected cells (Ferrero et al., 1998).

Vascular smooth muscle cells (VSMC) have an important role in vessel formation and therefore in the development and progression of cardiovascular diseases. Vitisin B, a resveratrol tetramer was shown to inhibit basal and platelet derived growth factor-induced VSMC migration. Strikingly, it did not inhibit VSMC proliferation but in contrast enhanced cell-cycle progression (Ong et al., 2011). In addition resveratrol was shown to inhibit VSMC proliferation through a block on G1-S phase and by an increase in apoptosis (Poussier et al., 2005). The effect of resveratrol on early signaling cascades in rat aortic VSMCs triggered by ANG II and epidermal growth factor (EGF) was investigated and the results showed that resveratrol does not influence ANG II-mediated transactivation of EGF-receptor but potently inhibits EGF-induced phosphorylation of Akt kinase, suggesting that resveratrol acts downstream of EGF-receptor transactivation in VSMCs (Haider et al., 2005). However, several other mechanisms are thought to be involved in the smooth muscle cell response. Resveratrol was tested on cultured human aortic smooth muscle cells (HASMC) in terms of growth and specific gene responses. Suppression of HASMC proliferation by resveratrol was accompanied by a dose-dependent increase in the expression of tumor suppressor gene p53 and heat shock protein HSP27 (Wang et al., 2006).

Platelets are centrally important in homeostasis and pathological conditions such as thrombosis, atherosclerosis, and inflammation and are therefore key targets in the treatment of cardiovascular disease. Thrombosis is the formation of a thrombus within the blood vessel resulting in occlusion of blood flow. It is a major problem that triggers both myocardial infarction and stroke. Targeting platelet aggregation using anti-platelet therapies is recognized as effective in the treatment and prevention of cardiovascular disease. Resveratrol, significantly inhibited collagen-, thrombin-, and ADP-induced aggregation of platelets from healthy subjects. Hypercholesterolemia induced by a high-cholesterol diet enhanced ADP-induced platelet aggregation; resveratrol inhibited this platelet aggregation induced by ADP *in vivo* in the absence of changes in serum lipid levels (Wang et al., 2002). Also polyphenolic grape extract induced a dose-dependent inhibition of trial to reduce alloimmunization to platelets (TRAP)-induced and ADP-induced platelet aggregation and Ca^2+^ mobilization; this inhibition was accompanied by activation of platelet endothelial cell adhesion molecule-1 (PECAM-1; de Lange et al., 2007).

## The Effects of Resveratrol in Animal Models of Cardiovascular Diseases

Supplemental resveratrol was shown to positively modify cardiovascular risk factors including body mass index, cholesterol, glucose tolerance, and systolic pressure in the Yorkshire swine hypercholesterolemic model (Robich et al., 2010).

In the rat ischemia and ischemia-reperfusion model, resveratrol showed protective effects against ischemia-reperfusion-induced arrhythmias and mortality. Resveratrol pretreatment both reduced the incidence and duration of ventricular tachycardia and ventricular fibrillation. During the same period, resveratrol pretreatment also increased NO and decreased lactate dehydrogenase levels in the carotid blood (Hung et al., 2000).

Wang et al. (2005b) determined whether resveratrol has alcohol-independent effects, and the size, density, and mean area of atherosclerotic plaques, and thickness of the intima layer were significantly reduced in hypercholesterolemic rabbits given dealcoholized red wine, red wine, or resveratrol. Collagens (COL1A, COL3A), lipoprotein lipase (LPL), and fatty-acid binding proteins (FABPs) involved in cardiovascular diseases and lipid metabolisms were upregulated by a high-fat diet and downregulated by resveratrol. Reverse transcriptase polymerase chain reaction confirmed that resveratrol and resveratrol-containing grape extract prevented the induction of FABP4 in peripheral blood mononuclear cells in female pigs fed a high-fat diet (Azorin-Ortuno et al., 2012).

Hypocholesterolemic properties of resveratrol in apo E(−/−) mice were investigated. The concentration of total-cholesterol (total-C) and LDL-cholesterol (LDL-C) in plasma was significantly lower in the resveratrol-supplemented groups compared to the control group. Plasma paraoxonase (PON) activity was significantly higher in the resveratrol group. The hepatic hydroxymetylglutaryl CoA (HMG-CoA) reductase activity was significantly lower in resveratrol group than in the control group. Resveratrol supplementation attenuated the presence of atherosclerotic lesions and periarterial fat deposition in the apo E(−/−) mice. The presence of ICAM-1 and VCAM-1 in atherosclerotic vessels was diminished in the resveratrol-supplemented apo E(−/−) mice (Do et al., 2008). Moderate consumption of red wine was shown to improve blood flow recovery by 32% after hindlimb ischemia in hypercholesterolemic ApoE-deficient mice. The number of endothelial progenitor cells, have an important role in postnatal neovascularization, was found as increased by 60% in ApoE mice exposed to red wine. Moreover, the migratory capacity of endothelial progenitor cells was significantly improved in red wine-drinking mice (Lefevre et al., 2007). The effect of resveratrol on the atherothrombotic tendency was assessed in apoE(−/−)/LDLR(−/−) mice and the formation of atheroma in the aorta and the size of laser-induced thrombus that mostly consisted of platelet aggregates was significantly reduced in the resveratrol fed group (Fukao et al., 2004).

Resveratrol was shown to significantly reduce infarct size compared with control in wild-type mouse hearts and this protection was shown through activation of the newly discovered survivor activating factor enhancement (SAFE) prosurvival signaling pathway that involves the activation of TNFα and the signal transducer and activator of transcription 3 (STAT3; Lamont et al., 2011).

## Clinical Data on Cardiovascular Diseases

Several different approaches have been proposed for the treatment of cardiovascular diseases. Understanding the mechanisms underlying these diseases is necessary to develop therapeutic interventions. Several epidemiologic studies tested the relationship between wine consumption and cardiovascular risk in order to investigate the effects of resveratrol. Low to moderate wine intake was shown to decrease the risk of death from cardiovascular diseases in a study involving almost 25,000 Danish subjects (Gronbaek et al., 1995). Another large population-based cohort study compared light to moderate wine drinkers to non-drinkers and reported that drinkers had less risk of death from all causes (Gronbaek et al., 2000). Renaud et al. (1999) showed that mortality from all causes was reduced by moderate wine consumption in 36,250 French middle-aged men, in an 18 years study. In 1,28,934 adults from Northern California, followed for 20 years, light to moderate wine intake decreased the risk of mortality from CHD (Klatsky et al., 2003). However, heavy drinking caused a high risk of mortality from non-cardiovascular reasons. Thun et al. carried out a large prospective study and observed the long-term effect of alcohol consumption in 4,90,000 men and women. The cardiovascular risk was 40% lower in men and women who had at least one drink daily (Thun et al., 1997). In addition acute intake of dealcoholized red wine was documented to improve endothelial function such as decrease in arterial stiffness in a number of studies (Karatzi et al., 2005). Also alcohol-free red wine decreased adverse effects of smoking on systolic blood pressure (Papamichael et al., 2006). 250–500 ml of acute dealcoholized red wine intake was found to improve brachial artery flow-mediated vasodilation (Agewall et al., 2000; Hashimoto et al., 2001).

As the main compound in red wine, resveratrol alone was also tested in the clinic. In 1997s, clinical trials were at the beginning and therefore the effects of long-term resveratrol supplementation was not known (Gehm et al., 1997). However, recent studies did not show any important side effect in the human trials (Boocock et al., 2007). Also recent clinical trials have observed resveratrol to be safe and well-tolerated at doses of up to 5 g/day. But the future possible side effects may bring limitations for these doses. When searched in the website of clinical trials (http://clinicaltrials.gov/ct2/results?term=resveratrol), totally 20 studies come up and only 5 of them are completed. These trials include the patients of cardiovascular diseases, type 2 diabetes, obesity, Alzheimer’s disease, and cancer. Some studies also tested the pharmacokinetic properties of resveratrol. A summary of published clinical trials can be found in Table 1.

**Table 1:** **Clinical data and trials of resveratrol-containing interventions (Patel et al., 2011)**.

Group	Resveratrol dose and application route	Results	Reference
25,000 male and female	Low-moderate wine intake	Decrease in cardiovascular risk	Gronbaek et al. (1995)
36,250 middle-aged men	Moderate wine intake for 18 years	Decrease in death from all causes	Renaud et al. (1999)
1,28,934 adults	Light-moderate wine intake for 20 years	Decrease in death from cardiovascular heart disease	Klatsky et al. (2003)
4,90,000 men and women	Long-term alcohol consumption (one drink daily)	Decrease in cardiovascular risk	Thun et al. (1997)
Male and female colorectal cancer patients	Caplets	Modulation of enzyme systems involved in carcinogen detoxification	Chow et al. (2010)
Healthy males and females	Wine and fruit juice	Pharmacokinetic and metabolic profile has been tested	Urpi-Sarda et al. (2005), Vitaglione et al. (2005), Ortuno et al. (2010), Goldberg et al. (2003), Meng et al. (2004)
Healthy males and females	Capsules	Pharmacokinetic and metabolic profile has been tested. No side effects have been observed.	la Porte et al. (2010), Boocock et al. (2007), Almeida et al. (2009), Nunes et al. (2009), Kennedy et al. (2010)

## Longevity

Resveratrol is believed to be good for increasing the lifespan and also for healthy aging. There are a lot of studies which tested the role of resveratrol in aging. Most interesting results came out from the lifespan studies in fruit flies and nematodes (Howitz et al., 2003; Bass et al., 2007). In 2003, Howitz and Sinclair showed that resveratrol extends the lifespan of *Saccharomyces cerevisiae* significantly (Howitz et al., 2003). Later the role of resveratrol on the lifespan of the worm *Caenorhabditis elegans* and the fruit fly *Drosophila melanogaster* was reported (Wood et al., 2004). Gruber et al. (2007) also showed that resveratrol has a positive effect on the lifespan of *C. elegans*. Furthermore resveratrol was observed to extend the lifespan of a short-lived fish *Nothobranchius furzeri* (Valenzano et al., 2006). In these organisms, lifespan extension depends on Sir2, a conserved deacetylase, and Sir2 underlies the beneficial effects of caloric restriction. Resveratrol produces changes associated with longer lifespan, including increased insulin sensitivity, reduced insulin-like growth factor-1 (IGF-I) levels, increased AMP-activated protein kinase (AMPK), and peroxisome proliferator-activated receptor-gamma coactivator 1α (PGC-1α) activity, increased mitochondrial number, and improved motor function (Baur et al., 2006). On the other hand, resveratrol treatment had only beneficial effects on elderly mice and did not increase the lifespan when started to be given in midlife (Pearson et al., 2008).

Resveratrol is implicated in many pathways related to stress conditions, diseases, and aging. Among them sirtuin (SIRT) pathway acts on the cell cycle, DNA damage response, metabolism, apoptosis, and autophagy with its deacetylase activity (Satoh et al., 2010). There are seven human SIRTs (SIRT1-7), and SIRT1 is implicated in the downstream pathways of calorie restriction to reduce the incidence of age-related diseases (Pacholec et al., 2010). Resveratrol is the most potent natural compound able to activate SIRT1 and the mechanism of resveratrol shows similar properties with the calorie restriction. However the certain mechanism of resveratrol remains unclear. In a recent work by Park et al. (2012) resveratrol was shown to inhibit cAMP-degrading phosphodiesterases and to increase NAD^+^ and activity of SIRT1.

Resveratrol was also shown to inhibit expression of the replicative senescence marker INK4a in human dermal fibroblasts, and microarray experiments showed that 47 of 19,000 genes were differentially expressed. These included genes for growth, cell division, cell signaling, apoptosis, and transcription. Genes involved in Ras and ubiquitin pathways, Ras-GRF1, RAC3, and UBE2D3, were downregulated. These changes suggest resveratrol might alter SIRT-regulated downstream pathways, rather than SIRT activity. Serum deprivation and high confluency caused nuclear translocation of the SIRT1-regulated transcription factor FOXO3a (Stefani et al., 2007).

Diminished mitochondrial oxidative phosphorylation and aerobic capacity are associated with reduced longevity and resveratrol is known to have impact on these parameters. Treatment of mice with resveratrol significantly increased their aerobic capacity, as evidenced by their increased running time and consumption of oxygen in muscle fibers. The effects of resveratrol were associated with an induction of genes for oxidative phosphorylation and mitochondrial biogenesis and were largely explained by a resveratrol-mediated decrease in PGC-1α acetylation and an increase its activity. This mechanism is consistent with resveratrol as a known activator of the protein deacetylase, SIRT1, and by the lack of effect of resveratrol in SIRT1(−/−) mouse embryonic fibroblasts (Lagouge et al., 2006). In another study, resveratrol was shown to inhibit the mitochondrial respiratory chain through complexes I–III in the rat brain (Zini et al., 1999).

## Resveratrol in Cancer

Cancer is one of the most destructive diseases worldwide. In the year 2000, cancer was suggested to be responsible for 12% of the nearly 56 million deaths worldwide and it is expected that cancer rates will further increase by 50% to 15 million new cases in the year 2020, mainly due to steadily aging populations in both developed and developing countries (Stewart and Kleihues, 2003).

Medical literature is full of studies showing the role of polyphenolic compounds and resveratrol in cancer, and resveratrol was especially shown to have chemopreventive and chemotherapeutic effects on different cancer types. Resveratrol may block carcinogen activation and increase detoxification via inhibition of phase I and induction of phase II enzymes. It may modulate cell cycle and induce apoptosis. Anti-angiogenic effects and suppression of invasion and metastasis is another important aspect of resveratrol. On the other hand, it may sensitize tumor cells for chemotherapy with other agents (Kundu and Surh, 2008).

**Figure 3:**
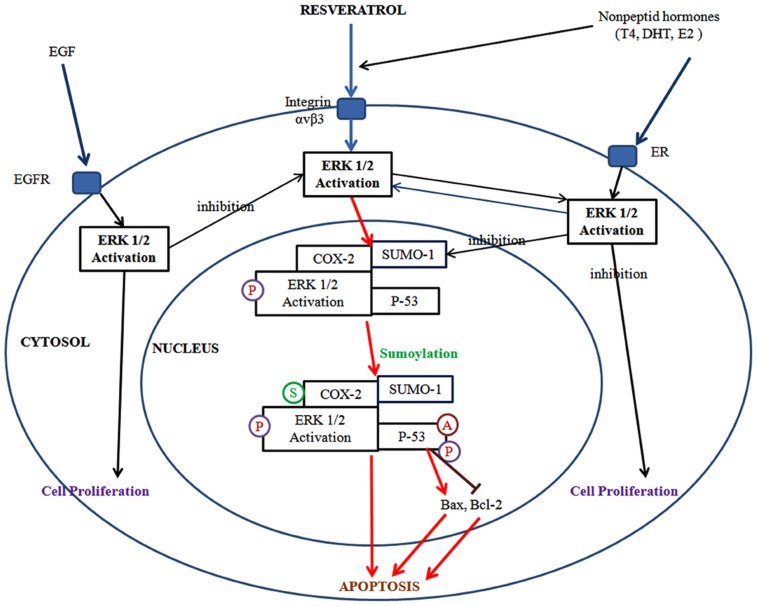
**Resveratrol binds to integrin αvβ3 and activates ERK1/2 regulates cyclooxygenase-2 (COX-2) expression**. COX-2 is associated with small ubiquitin-related modifier 1 (SUMO-1), and phosphoERK1/2 translocates to the nucleus where the complex binds to p53. In the nucleus, COX-2 is sumoylated (S), p53 is phosphorylated (P) and acetylated (A). The COX-2 and p53 complex binds to promoters of p53-responsive genes and initiates transcription. Activation of ERK1/2 and cell proliferation can be promoted by the binding of thyroid hormone and DHT each other to discrete sites on integrin αvβ3. Estrogen (E2) binds to estrogen receptor (ER) which is the cell surface receptor and integrin αvβ3 may assist in that process. Meanwhile, resveratrol and non-peptide hormone play a distinct role on the activation of ERK1/2 which differs from each other.

In this direction, resveratrol can influence important cellular and molecular mechanisms related to carcinogenesis such as inhibition of key proteins in signal transduction pathways such as mitogen-activated protein kinases, activator protein-1 (AP-1), and NFκB. It also affects cell-cycle regulation, apoptosis (Fresco et al., 2006), cell differentiation and proliferation, immune response, and chemical metabolism (Nichenametla et al., 2006; Figure 3). In this direction, Jang et al. (1997) first demonstrated the chemopreventive effects of resveratrol in multi-stage carcinogenesis (e.g., initiation, promotion, and progression also angiogenesis and metastasis).

In this part of the review, the effect of resveratrol on cancer will be described step by step including the effects on proliferation and transformation of cancer cells, angiogenesis, and therefore metastasis.

Apoptosis, a regulated form of cell death, is a complex process involving active participation of affected cells in a self-destruction cascade and inhibition of apoptosis leads to uncontrolled cell proliferation, resulting in events related to autoimmune disorders and cancer (Saikumar et al., 1999; Watson et al., 2000). Apoptosis is conceivably the most potent defense against cancer. Tumor cells can acquire resistance to apoptosis, for instance, by over expressing anti-apoptotic proteins such as Bcl-2 or down-regulate/mutate proapoptotic proteins such as Bax, the expression of both being regulated by the p53 tumor suppressor gene (Miyashita et al., 1994; Wang, 1999). P53 is a transcription factor essential for the prevention of cancer formation and it can be damaged by radiation, several chemicals, and viruses such as the human papilloma virus. The p53 pathway is not efficient in human cancer either by p53 gene mutation (60% of cancers) or by loss of cell signaling upstream and downstream of p53 (Bourdon, 2007).

Since apoptosis provides a physiologic mechanism for eliminating abnormal cells, dietary factors affecting apoptosis can present important effects on carcinogenesis. Conceivably, activation of apoptosis in pre-cancerous cells offers a prevention mechanism of cancer (chemoprevention) by dietary factors (Martin, 2006).

Subsequently, resveratrol has been shown to suppress proliferation of a wide variety of human tumor cells *in vitro* (Aggarwal et al., 2004; Howells et al., 2007; Kundu and Surh, 2008), which have led to numerous pre-clinical animal studies to evaluate the cancer chemopreventive and chemotherapeutic potential of resveratrol (Bishayee, 2009). There is growing *in vitro* evidence demonstrating the inhibitory effects of resveratrol on liver cancer. Delmas et al. (2000) showed that the proliferation of rat hepatoma and human hepatoblastoma HepG2 cells were negatively impacted by the addition of resveratrol to the culture medium and ethanol potentiated the effects of resveratrol in both cell lines. These results were attributed to the ability of resveratrol to prevent or delay the cells from entering mitosis and increasing the number of cells arrested in the S and G2/M phase. Hepatic growth factor (HGF) has largely been implicated in the ability of primary hepatic tumors to proliferate and invade adjacent tissue. The effects of resveratrol on HGF-mediated invasion were determined in HepG2 cells, in part, to understand the mechanisms of resveratrol’s anti-hepatocellular carcinoma property. Resveratrol was found to decrease HGF-induced scattering and invasion of liver cancer cells with concurrent inhibition of cell proliferation possibly due to a post-receptor mechanism rather than apoptosis (De Lédinghen et al., 2001).

Kuo et al. (2002) examined the anti-proliferative effects of resveratrol in two human liver cancer cell lines, namely HepG2 and Hep3B. The results showed that resveratrol inhibited cell growth only in p53-positive HepG2 cells, which was a result of cellular apoptotic death via p53-dependent pathway. It was also shown that resveratrol-treated cells were arrested in G1 phase and were associated with an increase in p21 and Bax expression. Michels et al. (2006) observed cytotoxic effect of resveratrol on rat hepatoma cells due to induction of apoptosis via caspase activation. Overexpression of anti-apoptotic Bcl-2 has been associated with elevated cyclooxygenase-2 (COX-2) expression (Tsujii and DuBois, 1995) and resveratrol was shown to suppress COX activity in the livers of mice treated with diethylnitrosamine (Khanduja et al., 2004).

Resveratrol induces p53-independent, X-linked inhibitor of apoptosis protein (XIAP)-mediated translocation of Bax to mitochondria where it undergoes oligomerization to initiate apoptosis. Resveratrol treatment promotes interaction between Bax and XIAP in the cytosol and mitochondria, suggesting that XIAP plays a critical role in the activation and translocation of Bax to mitochondria. This process does not involve p53 but requires accumulation of Bim and t-Bid in mitochondria. Bax primarily undergoes homo-oligomerization in mitochondria and plays a major role in release of cytochrome c to the cytosol. Bak, another key protein, that regulates the mitochondrial membrane permeabilization, does not interact with p53 but continues to associate with Bcl-xL. Thus, the proapoptotic function of Bak remains suppressed during resveratrol-induced apoptosis. Caspase-9 silencing inhibits resveratrol-induced caspase activation, whereas caspase-8 knockdown does not affect caspase activity, suggesting that resveratrol induces caspase-9-dependent apoptosis. These findings characterize the molecular mechanisms of resveratrol-induced caspase activation and subsequent apoptosis in cancer cells (Gogada et al., 2011).

Resveratrol can positively regulate SIRT1 transcription and activity, respectively. Increased SIRT1, in turn, inhibits expression and/or activity of several oncogenes, leading to reduced cell proliferation, increased apoptosis, and tumor suppression (Deng, 2009).

Lin et al. demonstrated the existence of a plasma membrane receptor for resveratrol near the arginine–glycine–aspartate recognition site on integrin αvβ3 that is involved in stilbene-induced apoptosis of cancer cells. Resveratrol treatment *in vitro* causes activation and nuclear translocation of mitogen-activated protein kinase, consequent phosphorylation of Ser-15 of p53, and apoptosis (Lin et al., 2011).

Resveratrol treatment of human glioblastoma cells was shown to induce a delay in cell-cycle progression during S phase associated with an increase in histone H2AX phosphorylation. Furthermore it was able to inhibit the ability of recombinant human TOPO II alpha to decatenate kDNA, so that it could be considered a TOPO II poison (Jo et al., 2006; Leone et al., 2010).

Cyclooxygenase-2 is an enzyme which has been found to be locally induced by proinflammatory mitogens, cytokines, and growth factors during inflammation and carcinogenesis. COX-2 has been associated with cell proliferation, differentiation, apoptosis, angiogenesis, invasiveness, and metastasis – all of which are involved in multi-stage tumorigenesis (Prescott and Fitzpatrick, 2000). Treatment with resveratrol was shown to decrease COX-2 expression in a concentration-dependent manner, suggesting involvement of anti-inflammatory mechanisms. Overexpression of anti-apoptotic Bcl-2 has been associated with elevated COX-2 expression (Tsujii and DuBois, 1995). Recently, it has been shown that resveratrol binds to COX-2 directly to inhibit its enzyme activity, suppresses COX-2-mediated PGE2 production, and exerts antitumor effects on human colon adenocarcinoma cells *in vitro* and *ex vivo*. According to prior *in vivo* studies, tumor-inhibitory effects of resveratrol in breast, esophagus, intestine, and skin have been associated with its ability to inhibit COX-2 (Bishayee, 2009). It was also found in the same study that resveratrol can decrease the degradation of IκB and the nuclear translocation of p65, indicating that this dietary agent can suppress diethylnitrosamine-mediated NFκB activation which is transcription factor for COX-2 in rat liver carcinogenesis.

ERK1/2 and MAPK isoforms that are inducible components of normal cellular signal transduction processes. Pathways of ERK1/2 activation can be triggered by a variety of stimuli and have been particularly well characterized in growth factor-stimulated cells or in the setting of inflammation. ERK1/2 is directly activated by MAPK-kinase (MEK1/2). Upstream activators in the MEK–MAPK pathway are Raf-1 kinase and, at the cell membrane, Ras (Malarkey et al., 1995). Resveratrol activates MAPK at low concentrations (1 pM to 10 μM), but higher concentrations (50–100 μM) of resveratrol can inhibit this signal transducing kinase in cancer cells (Miloso et al., 1999). It has been shown that resveratrol induces ERK1/2 activation in prostate (Lin et al., 2002; Shih et al., 2004), breast (Tang et al., 2006), glial (Lin et al., 2008b), head, and neck cancer cells (Lin et al., 2008a). The biochemical steps between formation of the COX-2-MAPK-ERK1/2 complex and activation of p53 in resveratrol-treated cells are now clear (Lin et al., 2008a,b). In fact, recent studies have demonstrated that posttranslational modification of COX-2 in the form of tyrosine phosphorylation regulates COX-2 activity in cerebral endothelial cells (Parfenova et al., 1998). Resveratrol-induced COX-2 associates with activated ERK1/2 in the nucleus of cancer cells (Lin et al., 2008a,b). Activated nuclear ERK1/2 has been shown to form complexes with transcriptionally active proteins, such as receptors for non-peptide hormones, signal transducing and activator of transcription (STAT) proteins, and the oncogene suppressor protein, p53 (Lin et al., 1999). Activated ERK1/2 in this context serves phosphorylation of (activation) the associated proteins.

Certain endogenous polypeptide growth factors, including EGF, IGF, and fibroblast growth factor, have been implicated in the development and progression of cancers (Djakiew, 2000; Shih et al., 2004). These factors are capable of rapidly activating ERK1/2 in several cell lines (Shih et al., 2004). EGF inhibits the action of resveratrol on both ERK1/2 activation and the induction of apoptosis in both androgen-responsive and unresponsive prostate cancer cells. These results suggest that increased ambient EGF levels would oppose any clinical actions that resveratrol may have.

Additionally, on the cancer cell surface, resveratrol binds to integrin αvβ3 and activates ERK1/2. In the same pathway, resveratrol induces COX-2 expression is also regulated by ERK1/2. Following the synthesis, COX-2 is associated with SUMO-1, and phosphoERK1/2 translocates to the nucleus where the complex binds to p53. In the nucleus, COX-2 is sumoylated, p53 is phosphorylated and acetylated. The COX-2 and p53 complex binds to promoters of p53-responsive genes and initiates transcription. Activation of ERK1/2 and cell proliferation can be promoted by binding of thyroid hormone and dihydrotestesterone (DHT) each other to discrete sites on integrin αvβ3. Estrogen (E2) binds to estrogen receptor (ER) which is the cell surface receptor and integrin αvβ3 may assist in that process. Meanwhile, resveratrol and non-peptide hormones play a distinct role on the activation of ERK1/2 which differs from each other. For resveratrol-induced inhibition of apoptosis, the activation of ERK1/2 via non-peptide hormones is known to be critical. When EGF binds to EGF-receptor, ERK1/2 is activated and the activation of ERK1/2 by EGF directly inhibits resveratrol-induced ERK1/2. Thus, the inhibitory role for resveratrol results in ERK1/2 downstream but EGF affects directly ERK1/2 activation (Lin et al., 2011; Figure 3).

On the other side, a significant amount of resveratrol accumulates and is retained in the liver (Bertelli et al., 1998; Vitrac et al., 2003; Abd El-Mohsen et al., 2006). Resveratrol has been shown to inhibit the hepatic carcinogen-activating enzymes, including cytochrome P450 1A1 (CYP1A1) and CYP3A/2 and induce hepatic phase 2 conjugating enzymes, namely NAD(P)H:quinine oxidoreductase, UDP-glucuronosyl-transferase, and glutathione S-transferase (GST) *in vitro* and *in vivo* (Ciolino et al., 1998; Canistro et al., 2009). The resultant effects of these enzyme modulations by resveratrol could be the reduced exposure of cells to carcinogens due to inhibition of carcinogen activation and/or elevated carcinogen detoxification and elimination. The most fascinating property of resveratrol, with regards to liver cancer, is its strong anti-inflammatory and antioxidant properties (Rubiolo et al., 2008), as both oxidative stress and inflammation have been strongly implicated in the occurrence and progression of hepatocellular carcinoma.

Resveratrol inhibited the development of preneoplastic lesions in carcinogen-treated mouse mammary glands in culture and inhibited tumorigenesis in a mouse skin cancer model (Jang et al., 1997). Athymic mice have been used for the determination of the role of resveratrol on human melanoma xenograft growth. The results suggested that resveratrol may not be useful in the treatment of melanoma and the effects of phytochemicals on cell cultures may not translate to the whole animal system which is thought to be because of the metabolization of resveratrol as will be explained below (Niles et al., 2006).

The role and the potential mechanism(s) of resveratrol were tested in *N*-nitrosomethylbenzylamine (NMBA)-induced rat esophageal tumorigenesis in F344 male rats. The results suggest that resveratrol suppressed NMBA-induced rat esophageal tumorigenesis by targeting COXs and PGE_2_ (Li et al., 2002). Antileukemic activity of resveratrol was examined *in vitro* and *in vivo* using a mouse myeloid leukemia cell line (32Dp210). Despite strong anti-proliferative and proapoptotic activities of resveratrol against the cells *in vitro*, a potential antileukemia effect occurs only in a small fraction of mice (Gao et al., 2002). Resveratrol was found to reduce the tumor volume, tumor weight, and metastasis to the lung in mice bearing highly metastatic Lewis lung carcinoma (LLC) tumors. The antitumor and antimetastatic activities of resveratrol was suggested to be due to the inhibition of DNA synthesis in LLC cells and the inhibition of LLC-induced neovascularization and tube formation (angiogenesis) of HUVEC by resveratrol (Kimura and Okuda, 2001).

Accumulating evidence suggests that combinations of two or more compounds could be much more effective. Combinations of several chemotherapeutic drugs also offer the possibility of lowering their doses and consequently may reduce unwanted adverse effects. Resveratrol has been found to potentiate the effects of chemotherapeutic agents and ionizing radiation (Cucciolla et al., 2007). Angiogenesis is a critical issue for the progression and severity of cancer. Resveratrol exerts anti-angiogenic activity (Chen et al., 2006; Garvin et al., 2006; Hu et al., 2007) and this activity has been related to the modulation of tumor cell release of thrombospondin-1 (TSP1) and vascular endothelial growth factor (VEGF). These effects lead to vascular endothelial cell apoptosis (Trapp et al., 2010). In human leukemia cells, secretion of VEGF was shown to be inhibited by resveratrol (Tang et al., 2007). Resveratrol inhibited pro-MMP-9 production at 25 or 50 μM concentrations in colon cancer cells (Kimura et al., 2008). Also expression and activity of MMP-2 and MMP-9 were downregulated in MCF-7 cells, mouse mammary tumors, and multiple myeloma cells (Banerjee et al., 2002; Sun et al., 2006; Tang et al., 2008).

Considering these advantages, resveratrol may be used in combination with other chemotherapeutic drugs and radiation therapy to enhance their therapeutic efficacy while limiting chemotherapy- and radiotherapy-associated negative side effects. Additionally, it seems to be logical to extend this approach to the field of chemoprevention, especially if one considers that dietary chemopreventive agents are naturally present in the diet in combination (Francy-Guilford and Pezzuto, 2008).

## Resveratrol in Neurodegenerative and Other Diseases

Resveratrol was claimed to be an ideal compound for the treatment of neurodegenerative diseases (Anekonda, 2006). Resveratrol was shown to diminish neurodegeneration related plaque formation in a region specific manner. The largest reductions in the percent area occupied by plaques were observed in medial cortex (−48%), striatum (−89%), and hypothalamus (−90%; Karuppagounder et al., 2009). Resveratrol was shown to significantly prevent intracerebroventricular (ICV) streptozotocin (STZ) induced cognitive impairment in rats (Sharma and Gupta, 2002). 3-Nitropropionic acid, an inhibitor of complex II of the electron transport chain, causes Huntington’s disease-like symptoms in rodents and resveratrol, with cyclooxygenase I inhibitory activity, significantly improved motor and cognitive impairment in the 3-nitropropionic acid-induced model of Huntington’s disease. These results were correlated with its antioxidant activity (Kumar et al., 2006). Resveratrol was shown to protect the spinal cord from secondary spinal cord injuries via improving the energy metabolism system and inhibiting the lipid peroxidation in the local injured spinal cord (Yang and Piao, 2003). The effect of chronic treatment of resveratrol was evaluated in focal ischemia induced by middle cerebral artery occlusion in rats. Treatment prevented motor impairment, caused a rise in levels of malondialdehyde (MDA) and reduced glutathione and the volume of infarct as compared to control (Sinha et al., 2002).

Hypercholesterolemia is a known risk factor for Alzheimer’s disease, and oxidative stress may play an important role in the progression of this disease. MDA levels as an indicator of oxidative stress in hypercholesterolemic rabbits were shown to be increased consistent with the previous results (Ozer et al., 1998, 2006; Aytan et al., 2008). Additionally, slight increase in 4-hydroxynonenal (HNE)-proteins, 3-nitrotyrosinated proteins, and protein carbonyls were observed in hippocampus area of the rabbits. Resveratrol was effective to modulate multiple mechanisms in Alzheimer’s disease and it was shown to lower the levels of secreted and intracellular amyloid-β (Aβ) peptides produced from different cell lines. Resveratrol was suggested to decrease Aβ amounts. It has no effect on the Aβ-producing enzymes β- and γ-secretases, but promote instead intracellular degradation of Aβ via a mechanism that involves the proteasome which may be induced by oxidative stress (Marambaud et al., 2005).

Regarding ischemic brain injury, resveratrol inhibited voltage-activated potassium currents in rat hippocampal neurons (Gao and Hu, 2005). In PC12 cells, Aβ induces the degradation of cytoplasmic IκBα and increases the translocation of p65 to the nucleus. These processes were reversed when the cells were treated with resveratrol (25 μM), suggesting that NFκB, in addition to its upstream signal transduction, was affected by resveratrol treatments (Jang and Surh, 2001).

Studies showed that resveratrol may be effective in diabetes. It was shown to possess hypoglycemic and hypolipidemic effects in STZ-induced type-1 diabetic rats by amelioration of common symptoms, such as body weight loss, polyphagia, and polydipsia. Also glucose uptake by hepatocytes, adipocytes, and skeletal muscle and hepatic glycogen synthesis were all stimulated by resveratrol treatment (Su et al., 2006). Estradiol is known to modulate insulin sensitivity and, consequently, glucose homeostasis. Insulin dependent and independent glucose uptake stimulation by resveratrol is shown to be regulated by ER (Deng et al., 2008). In STZ-nicotinamide-induced experimental type 1 diabetes in rats resveratrol resulted in significant decrease in the levels of blood glucose, glycosylated hemoglobin, blood urea, serum uric acid, serum creatinine, and diminished activities of aspartate transaminase (AST), alanine transaminase (ALT), and alkaline phosphatase (ALP). The antihyperglycemic nature of resveratrol is also evidenced from the improvement in the levels of plasma insulin and hemoglobin (Palsamy and Subramanian, 2008). Diabetic nephropathy is a serious microvascular complication and one of the main causes of end-stage renal disease. Treatment with resveratrol significantly attenuated renal dysfunction and oxidative stress in diabetic rats (Sharma et al., 2006). The effects of resveratrol on memory and on acetylcholinesterase (AChE) activity in the cerebral cortex, hippocampus, striatum, hypothalamus, cerebellum, and blood in STZ-induced diabetic rats were tested. In this study, resveratrol prevented the impairment of memory induced by diabetes and the increase in AChE activity (Schmatz et al., 2009). Insulin provides blood glucose homeostasis by the stimulation of glucose uptake by translocation of glucose transporter Glut-4 from intracellular pool to the caveolar membrane system. Resveratrol-mediated Glut-4 translocation in the STZ-induced diabetic myocardium was tested and resveratrol was shown to trigger some of the similar intracellular insulin signaling components in myocardium such as eNOS, Akt through AMPK pathway and also by regulating the caveolin-1 and caveolin-3 status that might play an essential role in Glut-4 translocation and glucose uptake in STZ- induced type-1 diabetic myocardium (Penumathsa et al., 2008).

In several studies, resveratrol has been shown to possess anti-inflammatory effects since it plays role as aryl hydrocarbon receptor antagonist and inducible COX-2 inhibitor. The effect of resveratrol was examined in a model of hyperalgesia induced by carrageenan in the rat. Pretreatment with resveratrol did not reverse swelling and edema, but reversed the hyperalgesia induced by local tissue injury provoked by carrageenan (Gentilli et al., 2001). Resveratrol was found to inhibit NO generation, cytosolic iNOS protein and steady-state mRNA levels in lipopolysaccaride (LPS) activated RAW 264.7 macrophages. In electrophoretic mobility shift assays, the activation of NFκB induced by LPS was inhibited by resveratrol (Tsai et al., 1999). In a study carried out in rabbits, arthritis was induced by intra-articular injection of LPS into the knee joints. Resveratrol was shown to ameliorate the synovial inflammation after intra-articular injection (Elmali et al., 2007).

Resveratrol adversely affects the critical early event in the herpes simplex virus types 1 and 2 (HSV-1 and HSV-2) replication cycle, that has a compensatory cellular counterpart (Docherty et al., 1999). Also topically applied resveratrol inhibits HSV lesion formation in the skin of mice (Docherty et al., 2004). Human cytomegalovirus replication was also inhibited by resveratrol via the blockage of virus-induced activation of the EGFR and phosphatidylinositol-3-kinase signal transduction as well as NFκB activation (Evers et al., 2004). Resveratrol was found to inhibit varicella-zoster virus replication in a concentration-dependent and reversible manner (Docherty et al., 2006). Resveratrol was shown to synergistically enhance the anti-HIV-1 activity of the nucleoside analogs zidovudine, zalcitabine, and didanosine by the reduction of viral replication (Heredia et al., 2000).

## Administration Doses, Routes, and Bioavailability of Resveratrol

Resveratrol synthesis in plants is catalyzed by resveratrol synthase enzyme. In red wine, the levels are much higher than in white wine because the skin of the grapes are removed during the fermentation of white wine (Gu et al., 1999). Also the levels in red wine depend on the type of the grape. Besides wine, peanut is another source of resveratrol (Wang et al., 2005a). Also mulberry is a source and is sold as nutritional supplement (Stewart et al., 2003). Cocoa and dark chocolate include low levels of resveratrol (Hurst et al., 2008). And recently Japanese Knotweed has become popular for its high content of a resveratrol analog, piceatannol (Piotrowska et al., 2012).

Before clinical trials, researchers use *in vitro* results to be able proceed. Choosing the right concentration is a crucial problem for the *in vitro* studies. The tested concentration should reflect the clinical usage and should be estimated according to the target pathway. Generally used concentrations of resveratrol range from μmol/l to mmol/l and observed metabolites are usually in nmol/l concentrations. Elevated doses are sometimes used to force the outcome but these results should be carefully interpreted (Scott et al., 2012).

*In vitro* studies have used several different concentrations of resveratrol according to the target of the study. Chao et al. (2010) showed that 0.1, 1, and 10 μM resveratrol were neuroprotective in SH-SY5Y cells as shown by lactic dehydrogenase release, caspase-3 activity, and also by JNK and mTOR pathway studies. In another study in the same cell line, a 50 μM concentration was shown to reduce cellular death by inhibiting the activation of caspase 7 and the degradation of PARP (Nicolini et al., 2001). Further, resveratrol at 171–342 μg/ml concentrations was shown to be anti-microbial against *Staphylococcus aerius*, *Enterococcus faecalis*, and *Pseudomonas aeruginosa* (Chan, 2002).

For the cancer chemopreventative action of resveratrol, it is important to know the pathway that is effective. Generally, proliferation of the cells have been shown to be suppressed above 5 μM resveratrol which may often be close to 100 μM (Scott et al., 2012). Resveratrol (52–74 μM) was shown to antagonize the effect of linoleic acid, a potent breast cancer cell stimulator, and suppressed the growth of both ER-positive and -negative cell lines ((Damianaki et al., 2000; Nakagawa et al., 2001). 10–40 μM were shown to be enough to induce apoptosis in JB6 mouse epidermal cells via JNK activation (She et al., 2002).

Regarding its cardioprotective role, NOS expression was shown to increase at 1 μM concentration while it was decreased at 60 nM (Gu et al., 2006).

There are several routes of administration for resveratrol. Buccal application is one way, this occurs by direct absorption through mucous membrane of the mouth. However, the low solubility of resveratrol limits the absorbable amount through the buccal mucosa. Oral application as a pill restricts the bioavailability because resveratrol is metabolized rapidly into glucuronate and sulfonate conjugates in the intestine and liver (Walle et al., 2004). Following oral administration in a dose of 25 mg, only trace amounts of unchanged resveratrol (<5 ng/ml) were detected in plasma. Although the systemic bioavailability of resveratrol is very low, accumulation of resveratrol and potentially active resveratrol metabolites in epithelial cells along the aerodigestive tract is thought to still produce cancer-preventive and other effects (Walle et al., 2004). On this basis, SRT-501 and later SRT2104 and SRT2379 have been developed as different formulations of resveratrol.

Several pharmacokinetic studies have been carried out to test the bioavailability of resveratrol. In rat studies, *trans*-resveratrol was found to be in different conjugates throughout the body and its aglycone form was found to be the main metabolite retained in the tissues (Marier et al., 2002; Wenzel et al., 2005; Abd El-Mohsen et al., 2006). In another study, abundant *trans*-resveratrol-3-*O*-glucuronide and *trans*-resveratrol-3-sulfate were identified in rat urine, mouse serum, and in incubated rat and human hepatocytes (Yu et al., 2002).

The steady-state pharmacokinetics and tolerability of *trans*-resveratrol 2000 mg given twice daily with food, quercetin and alcohol (ethanol) was tested in eight healthy subjects. This resulted in adequate exposure and was well tolerated, although diarrhea was frequently observed. In order to maximize the actions of *trans*-resveratrol, it should be taken with a standard breakfast and not with a high-fat meal. Furthermore, combined intake with quercetin or alcohol did not influence *trans*-resveratrol efficiency (la Porte et al., 2010).

A phase I study of oral resveratrol (single doses of 0.5, 1, 2.5, or 5 g) was conducted in 10 healthy volunteers and consumption of resveratrol at these levels did not cause serious adverse affects (Boocock et al., 2007).

Chemopreventive parameters and metabolic conversion of resveratrol were tested *in vivo*. Feeding of different dosages of resveratrol revealed no effect on the different chemopreventive parameters, and the formation of *trans*-resveratrol-3-sulfate, *trans*-resveratrol-4′-sulfate, *trans*-resveratrol-3,5-disulfate, *trans*-resveratrol-3,4′-disulfate, *trans*-resveratrol-3,4′,5-trisulfate, *trans*-resveratrol-3-*O*-beta-d-glucuronide, and resveratrol aglycone was detected by HPLC analysis, depending on the biological material. The lack of effect of resveratrol on the chemopreventive parameters is probably due to the formation of various conjugates reducing its bioavailability in the rat (Wenzel et al., 2005).

## Conclusion

The enormous amount of clinical and animal studies suggests that resveratrol may have beneficial effects to improve health, prevent, and/or treat chronic diseases in humans. However, despite a large amount of circumstantial and experimental evidence, definitive clinical studies are largely missing. Moreover, explaining the molecular basis of the beneficial results of resveratrol still awaits further detailed and extensive epidemiological studies. Further studies are needed regarding the genetic factors that account for differences in bioavailabity and physiological responses to resveratrol between individuals. It should be clarified whether resveratrol can have either synergistic or additive effects in combination with other therapies.

## Conflict of Interest Statement

The authors declare that there search was conducted in the absence of any commercial or financial relationships that could be construed as a potential conflict of interest.
